# Primary ovarian hydatid cyst in a postmenopausal woman: A rare case report

**DOI:** 10.1016/j.ijscr.2020.03.006

**Published:** 2020-03-07

**Authors:** Taxiarchis Katsamagkas, Ioannis Tsakiridis, Dimitrios Evaggelinos, Paraskevi Skafida, Themistoklis Dagklis, Ioannis Kalogiannidis

**Affiliations:** a Department of Obstetrics and Gynaecology, Interbalkan Medical Center of Thessaloniki, Greece; b Third Department of Obstetrics and Gynaecology, Faculty of Medicine, Aristotle University of Thessaloniki, Greece

**Keywords:** Echinococcus, Hydatid cyst, Primary, Ovary

## Abstract

**Introduction:**

Hydatid cysts of the female reproductive system represent a rare entity. We report a case of a primary hydatid cyst of the right ovary of a postmenopausal woman that was diagnosed incidentally.

**Presentation of the case:**

A 72-year old overweight woman with history of three vaginal deliveries was referred due to uterine prolapse. During clinical examination of the patient, preoperative pelvic ultrasound revealed an ovarian cyst with benign characteristics. After oncology review, a laparoscopically assisted vaginal hysterectomy with bilateral salpingo-oophorectomy was conducted; the histopathology of the cyst revealed a hydatid cyst. Postoperative abdominal computed tomography identified no other sites of infection, indicating the ovary as the primary site of infection. During follow-up no recurrence occurred.

**Discussion:**

Hydatid cysts usually have a low progression and tend to be asymptomatic. Medical treatment may be successful in small asymptomatic cysts, however a close follow-up is needed after antihelminthic treatment. Surgical management with removal of the intact cyst is the preferred method for large cysts.

**Conclusion:**

We report a rare case of ovarian hydatid cyst that was effectively removed intact during a laparoscopically assisted vaginal hysterectomy with no recurrence during follow-up.

## Highlights

• Primary hydatid cysts of the ovaries represent a very rare entity that may be diagnosed incidentally.
• Ultrasound is the gold standard diagnostic method for ovarian hydatid cysts.
• Medical treatment may be successful in small asymptomatic cysts, however surgical management with removal of the intact cyst is the preferred method for large cysts.

## 1 Introduction

The presence of hydatid cysts in the female reproductive system is a very rare entity and constitutes about one out of 200 hydatid cysts [1,2]. So far, only a few such cases have been reported in the literature [3]. Despite the fact that accurate detection is important, a conclusive diagnosis of echinococcosis cannot be usually confirmed preoperatively. Additionally, since the liver and the lung are the most common locations of this parasitic infection [4], it is extremely rare for an ovary to be the primary site. Hence, we report a case of a postmenopausal woman with a primary ovarian hydatid cyst, that was incidentally diagnosed. This case report has been written in accordance to the SCARE criteria [5].

## 2 Presentation of the case

A 72-year-old woman was referred to the outpatient gynecologic department due to suspected uterine prolapse. She had a history of three vaginal deliveries, did not smoke and reported menopause at the age of 52. She was treated with valsartan for chronic hypertension, while she reported no history of jaundice or surgical operations. At the age of 62 she was diagnosed with an abnormal Pap test (low-grade squamous intraepithelial lesion - LSIL) with negative HPV DNA test. However, during follow-up, no subsequent cervical pathology was detected.

**Fig. 1:**
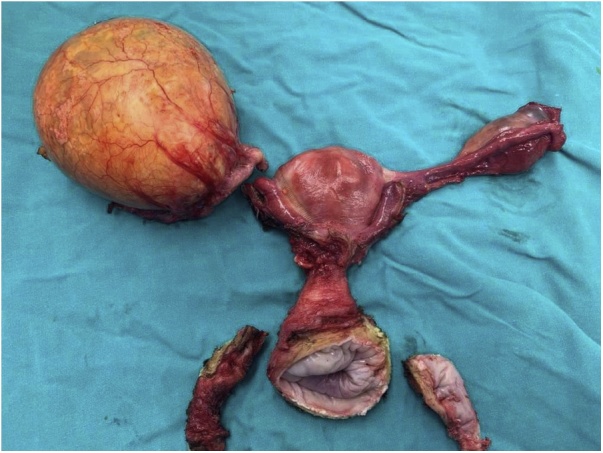
Postoperative specimen of the uterus, including the right ovary with the hydatid cyst and the left adnexa.

On the current physical examination, uterine prolapse stage III (POP-Q) was confirmed. The body mass index of the patient was 28 kg/m^2^ and all vital signs were normal; the temperature was 36.2 °C, the heart rate was 68 beats/min, the blood pressure was 120/75 mmHg, the respiration rate was 18 breaths/min and O_2_ saturation was 100%. Serum liver and renal tests, as well as chest X-Ray findings were also normal. As part of a standard preoperative protocol, transvaginal sonography (4–9 MHz transducer) was performed. This identified a cystic multilocular tumor (max diameter 9 cm), with solid components (maximal diameter <7 mm) and presence of acoustic shadow under the right adnexa, while no abnormalities were detected in the left adnexa or the uterus. Serum CEA and Ca-125 values were normal. Using the International Ovarian Tumor Analysis (IOTA) ADNEX model, we calculated a low risk of malignancy 1.4% [6]. After an oncology review, a laparoscopic approach was decided. Thus, a laparoscopically assisted vaginal hysterectomy (with removal of the adnexa) was offered to the patient (Fig. 1). Intraoperatively, the abdomen and the pelvis were explored without any further abnormal findings. A frozen section study of the right adnexa ruled out malignancy and the estimated blood loss was 300 ml. During the next 3 days, all the vital signs were stable and the patient was discharged.

The histopathology specimen revealed a hydatid cyst of the ovary. Additionally, a postoperative abdominal Computed Tomography (CT) revealed no other sites of infection, including the liver; thus, the ovary was considered the primary site of infection. A referral to the outpatient department of Infectious Diseases was offered to the patient. During follow-up, to date, there was no recurrence of the disease.

## 3 Discussion

Echinococcosis is endemic in several countries of the world, including East Europe, South America and Mediterranean area [7]. Echinococcus granulosus is the most common parasitic worm, with a lifespan of five to 20 months. Echinococci grow in the intestinal tract, where they release their eggs; the latter are excreted with the feces and ingested by hosts, including humans [8]. A typical hydatid cyst consists of three layers; the external is called pericyst, consists of fibrous tissue and is usually thick in the liver hydatid cysts [9]. In our case the pericyst was thin, as typically in extrahepatic cysts.

As already mentioned, liver is the most common primary location for this disease [10]. However, primary extrahepatic echinococcosis, as in our case, has been reported in up to 2% of patients with abdominal hydatid disease [10]. Several other locations of the disease have been reported, including the abdominal wall, the omentum, the pancreas and the uterus [10,11].

Hydatid cysts typically have a slow progression and tend to be asymptomatic; sometimes jaundice may be the main symptom due to intrabiliary rupture [12]. Other signs and symptoms that have been reported in a series of patients include a palpable mass, urine hesitation and abdominal pain [10].

Regarding diagnosis, hydatid cysts of the ovaries often mimic ovarian tumors, leading that way to unnecessary diagnostic procedures [13]. Ultrasound is the gold standard method (sensitivity 93%), however CT has a higher sensitivity for the diagnosis of peritoneal disease (up to 100%) [14]. Serological tests may also be useful for an accurate diagnosis. Moreover, IgG against the parasite antigen with ELISA (sensitivity 95%) and indirect hemagglutination test may also be used for the diagnosis (sensitivity 87.5%) [15].

As for the management, asymptomatic cysts may be treated with antihelminthic drugs; albendazole is typically used in uncomplicated hydatid disease, but close monitoring is mandatory during treatment because of the possible side effects [10,16]. In cases of symptomatic or large cysts, surgery is the preferred treatment; The goal of surgery is to remove all the parasitic cysts, avoiding spillage, if possible [12]. In case of a cyst rupture during surgery, the fluid should be quickly removed, due to the high risk of anaphylaxis or recurrence [2]. Moreover, the World Health Organization recommends adjuvant chemotherapy and albendazole in cases of cyst rupture or residual disease after the surgery [17]. In our case, no such therapy was offered due as the removal was intact. After a successful surgical operation, there is a recurrence rate of 2%, while the survival rate is up to 95% [12].

## 4 Conclusion

This was a rare case of a primary ovarian hydatid cyst that was diagnosed incidentally following a referral due to uterine prolapse. This case highlights the need for higher awareness of rare types of ovarian masses. The healthcare provider should always keep in mind that, especially in cases of endemic hydatid disease, echinococcosis should always be included in the differential diagnosis of ovarian cysts, leading that way to earlier and more accurate diagnosis and associated management of the disease.

## Conflict of interest

The authors have no conflict of interest to declare.

## Funding sources

This research did not receive any specific grant from funding agencies in the public, commercial, or not-for-profit sectors.

## Ethical approval

This article does not contain any personal information that can lead to the identification of the patient.

## Consent

Written informed consent was obtained from the patient for publication of this case report and accompanying images. A copy of the written consent is available for review by the Editor-in-Chief of this journal on request.

## Author contribution

Katsamagkas Taxiarchis: Paper design, data collection, paper writing.

Tsakiridis Ioannis: Paper design, data collection, paper writing.

Evaggelinos Dimitrios: Paper design, paper writing.

Skafida Paraskevi: Paper review, Picture preparation.

Dagklis Themistoklis: Picture preparation, paper review.

Kalogiannidis Ioannis: Paper design, data collection, paper review.

## Registration of research studies

N/A.

## Guarantor

Ioannis Tsakiridis, Ioannis Kalogiannidis.

## Provenance and peer review

Not commissioned, externally peer-reviewed.
